# *Dolichocephalovirinae* Phages Exist as Episomal Pseudolysogens Across Diverse Soil Bacteria

**DOI:** 10.3390/microorganisms13061239

**Published:** 2025-05-28

**Authors:** Tannaz Mohammadi, Bert Ely

**Affiliations:** Department of Biological Sciences, University of South Carolina, Columbia, SC 29208, USA; tannaz@email.sc.edu

**Keywords:** lysogenic bacteria, phage-bacteria interactions, broad-host-range bacteriophages, microbial interactions, bacterial diversity, *Caulobacter*

## Abstract

Interactions between bacteria and bacteriophages are important for the maintenance of soil communities. In this study, we characterized the giant bacteriophages found within diverse soil bacteria and 14 additional phages isolated directly from soil samples. Based on their genome sizes and genetic composition, we concluded that these phages belong to the *Dolichocephalovirinae* subfamily. In addition, we used pulsed-field gel electrophoresis to show that the genomes of these phages were present as episomal pseudolysogens in the cytoplasm of their host cells. These findings suggest that episomal phages are important components of soil microbial ecosystems. Understanding the interactions between bacteriophages and bacteria is essential for microbial ecology, as they influence nutrient cycling, community composition, and host evolution. Furthermore, these phage-bacteria dynamics offer potential applications in plant disease control, as bacteriophages could serve as biocontrol agents against soilborne pathogens, promoting sustainable agricultural practices.

## 1. Introduction

Plant diseases significantly impact crop yields and quality, resulting in billions of dollars in losses annually in the United States [1,2]. These diseases can reduce productivity, degrade quality, and even contaminate food supplies. They are caused by various living organisms, including fungi, bacteria, viruses, and nematodes. Effective management of plant diseases is crucial for ensuring food security. Soil-dwelling organisms and microbes, such as insects, bacteriophages, bacteria, mites, and fungal vectors, play critical roles in either exacerbating or mitigating plant diseases. To develop effective disease control strategies, it is essential to understand the biology and interactions of these soil organisms [1,2,3,4]. Bacteriophages are key drivers of microbial community dynamics, influencing diversity, richness, abundance, evolution, and bacterial physiology within a given habitat [5,6,7]. Over the past two decades, they have been extensively studied for their roles in antimicrobial resistance, food safety, food processing, agriculture, environmental sustainability, and medicine [8,9,10,11]. Despite their recognized importance, much remains unknown about how phages regulate bacterial populations in complex natural ecosystems, particularly in soil environments [12,13,14,15]. While previous studies have largely focused on their lytic and lysogenic cycles, the ecological significance of pseudolysogeny and episomal phage persistence in shaping bacterial interactions is still poorly understood [16]. Addressing this gap is crucial for uncovering the broader implications of phage-bacteria interactions in microbial ecology and plant disease control.

Currently, our laboratory is focused on isolating bacteria and bacteriophages from soil and plant roots to explore their impact on plant-bacteria interactions. By studying these microorganisms, we aim to uncover their roles in influencing plant health and their potential applications in sustainable agriculture.

Bacteriophages, or phages, are viruses that infect bacteria. They are found wherever bacteria are present. Phage-host interactions in soil environments play a crucial role in shaping microbial communities and biogeochemical processes. One key mechanism through which phages influence their bacterial hosts is the transfer of auxiliary metabolic genes (AMGs), which can enhance host adaptation to environmental stresses. These AMGs may alleviate the metabolic burden of bacteria by supporting essential functions such as nutrient acquisition, stress resistance, and pollutant degradation [17]. In peatland soils, phage-encoded AMGs involved in carbohydrate metabolism contribute to carbon cycling, affecting microbial decomposition and nutrient availability [18,19]. Similarly, in paddy soils, AMGs associated with phosphorus and nitrogen cycling aid in nutrient uptake and transformation, influencing soil fertility [20]. In contaminated environments, phages can facilitate bacterial adaptation by transferring genes that enable pesticide degradation or confer heavy metal resistance, thereby enhancing microbial survival under toxic conditions [21,22]. These interactions demonstrate that phages are not just viral predators but also play an active role in microbial evolution and ecosystem stability. A deeper understanding of soil phage AMGs is essential for unraveling their impact on microbial metabolism, nutrient cycling, and environmental resilience.

Bacteriophages are generally classified into three lifestyles: virulent, temperate, and chronic [23]. Virulent phages, also known as lytic phages, employ the lytic cycle, infecting bacterial cells to rapidly replicate and produce new virions, ultimately lysing the host cell to release new phage particles. Temperate phages can also lyse host cells, but they have the unique ability to enter the lysogenic cycle. In this state, they remain dormant as prophages with their genomes integrated into the bacterial chromosome [23,24]. Prophages often benefit their bacterial hosts by carrying genes that provide advantages, such as virulence factors [25] or anti-phage defense systems [26]. They may even mediate competitive interactions between bacterial strains [27]. Chronic phages, such as filamentous phages from the *Inoviridae* family, adopt a different strategy. They continuously release virions from the infected host without causing cell death, enabling persistent infection and propagation [28]. This diverse range of lifecycles highlights the versatility and ecological significance of bacteriophages.

Categorizing the diverse alternative lifestyles of bacteriophages presents significant challenges. Terms such as “pseudolysogeny” and “carrier state” have been inconsistently used to describe these alternative phage-host interactions. The term “carrier state” typically refers to specific scenarios, such as those observed with P22 or ΦCrAss001, where phage and bacterial populations coexist at the community level. Within a bacterial population, some cells are resistant to a particular bacteriophage while others remain susceptible to infection. This division into resistant and susceptible subpopulations allows both the bacteriophage and the bacterial population to coexist over time. Resistant cells survive and are not killed by the phage, while susceptible cells are infected and may be lysed, but they also support phage replication. This balance prevents either the phage or the bacterial population from being completely eliminated, leading to stable coexistence at the population level [29,30].

In contrast, “pseudolysogeny” lacks a clear, consistent definition and is often used as a catch-all term for poorly understood or undefined phage-host interactions [16,24,29]. Ripp and Miller [24,31] defined pseudolysogeny as a distinct type of phage-host interaction where the phage genome neither integrates into the host chromosome as a prophage (lysogeny) nor initiates a lytic response. Instead, the phage nucleic acid remains inside the starved host cell in a dormant, non-active state as a plasmid-like form called an episome. Unlike lysogeny, in which the phage genome replicates in synchrony with the host chromosome, pseudolysogeny leads to asymmetrical inheritance of the phage genome. During cell division, only one daughter cell inherits the phage genome, while the other does not [24,31].

Ripp and Miller proposed that pseudolysogeny arises under extreme starvation conditions, where insufficient energy is available for the phage to initiate either a lytic or lysogenic infection cycle. They hypothesized that as environmental conditions improve and nutrients become more abundant, pseudolysogens can transition to either virion production (lytic cycle) or establish “true” lysogeny. Furthermore, they argued that pseudolysogeny likely plays a crucial role in natural ecosystems by maintaining phage genetic material over extended periods in unfavorable conditions, thereby enabling phages to survive and persist until conditions become more suitable for active infection [24,32]. Therefore, coexistence, including phenomena like lysogeny and pseudolysogeny, can provide conditional benefits to both the phage and the host. For the phage, residing within the host cell shields its genome from external environmental stressors, such as UV radiation, extreme temperatures, and pH fluctuations, ensuring its survival during unfavorable conditions [24,31,32]. For the host bacteria, the phage may provide some degree of immunity to infection by additional phages.

In a previous study performed on soil bacteria, most of the isolated strains were lysogenic or pseudolysogenic and could produce viable phages [33]. To identify additional phages, the supernatants from the remaining six bacterial strains were tested against all twenty-one bacterial strains isolated from the sample sites. Phages from four of the six strains, *Sphingomonas* strain RBW1, *Pseudomonas* strain RBW6, *Brevundimonas* strain RBW18, and *Caulobacter rhizosphaerae* strain RBW25, were able to lyse at least one of the twenty-one bacterial strains. In addition, we isolated and characterized 14 additional bacteriophages from soil samples. All the phages are giant phages with genome sizes around 200 kb, and we showed that these phage genomes exist in their host bacteria as episomes. Consistent with Lood et al. (2011) [34] and Wottrich et al. (2024) [35], we designate this variant of classical lysogeny as episomal pseudolysogeny since the phage genome is present in the host cytoplasm as a replicating episome in contrast to lysogeny where the phage genome has recombined with and integrated into the host genome.

## 2. Materials and Methods

### 2.1. Bacterial Culture Preparation

All bacterial cultures used in this study were grown in peptone yeast extract (PYE) liquid medium, which consisted of 0.2% peptone, 0.1% yeast extract, 0.5 mM CaCl_2_, and 0.8 mM MgSO_4_. Tubes containing 3 mL of PYE were inoculated with a single colony of the desired strain. For rapid growth, cultures were incubated overnight at 34 °C. Alternatively, for slower growth, they were incubated over the weekend at 24 °C.

### 2.2. Isolation of Bacteriophages from Bacterial Strains

To induce prophages in bacterial strains that did not produce phages during normal cell growth, 500 μL of fresh overnight bacterial culture was used to inoculate a flask containing 25 mL of PYE broth in a shaker at 30 °C and 60 rpm. After four hours, 24 μL of a Mitomycin C solution (12.5 mg/mL) was added to each flask, and the cultures were incubated overnight. The next morning, the cultures were centrifuged at 4000× *g* for 10 min and the supernatant was retained. Centrifugation was repeated until no visible bacterial pellets remained in the supernatant, ensuring the removal of most of the bacteria.

To eliminate the remaining bacteria, 1 mL of chloroform was added to the supernatant. The mixture was shaken vigorously and allowed to settle to ensure thorough bacterial disruption. After chloroform treatment, 10 μL of the treated supernatant was spotted onto fresh bacterial lawns seeded with 100 μL of a potential host strain in 3.5 mL of SSM (PYE plus 0.3% agar) on PYE agar plates. After overnight incubation, the plates were examined for the presence of plaques or lysis, which would confirm the presence of bacteriophages. If lysis was observed, the new phage was purified twice from single plaques before growth of the phage at a dilution that would produce overlapping plaques. After overnight incubation of the growth plate, 5 mL of PYE was added to the plate. Subsequently, the liquid was decanted into a test tube after allowing time for the phage to diffuse into the PYE, and a small amount of chloroform was added to kill contaminating bacteria. To preserve the new phages, 500 μL of each lysate was frozen at −70 °C for long-term storage.

### 2.3. Independent Isolation of Bacteriophages from Soil and Plant Root Samples

Soil and plant root samples were collected from various sites along Rocky Branch Creek, where Sumter St. intersects the creek on the University of South Carolina campus. To release soil-associated bacteriophages, soil and roots were separately soaked in sterilized tap water at 4 °C for 24 h.

A 10 mL aliquot of the soil-soaked suspension was filtered using a 0.45 μm filter to remove bacteria and particulate matter. The filtrate was then mixed with 50 μL of a culture of the streptomycin-resistant *Caulobacter* strain SC1004, 5X PYE broth, and 0.6 mg/mL streptomycin. The mixture was incubated at 30 °C on an orbital shaker overnight to allow phage propagation. The resulting phage-enriched suspension was transferred to a 15 mL sterile tube and centrifuged as described above.

To confirm the presence of bacteriophages, 10 μL of the chloroform-treated supernatant was spotted onto bacterial lawns prepared by mixing 100 μL of a potential host strain with 3.5 mL of SSM (PYE plus 0.3% agar) and plating on PYE agar. After overnight incubation, plates were examined for plaque formation, indicating the presence of bacteriophages. If plaques were observed, phages were purified by two rounds of single-plaque isolation, followed by growth and storage as described above.

### 2.4. Phage Genome Size

Phage genome size was determined through pulsed-field gel electrophoresis (PFGE). Agarose plugs containing phage DNA were prepared following the protocol outlined by Dingwall et al. [36]. Briefly, a mixture of phage lysate and 1% agarose in SBA (2.4 g boric acid, 0.4 g NaOH, pH 8 per liter) in a 1:1 ratio was drawn up into a 1 cc syringe and allowed to solidify. The resulting mixture was then sliced into 1 mm thin discs (plugs) and incubated overnight at 50 °C in 2 mL of lysis buffer (1.9 mL of 1% Sarkosyl in 0.5 M EDTA and 0.1 mL of proteinase K at 20 mg mL^−1^). After incubation, the plugs were washed twice with 2 mL of TE buffer and treated with 30 µL of PMSF (17.4 mg mL^−1^), and then three times with 2 mL of TE buffer. Following the wash steps, one of the plugs was subjected to PFGE at 6 V for 14 h, with an initial switch time of 6 s and a final switch time of 20 s, and phage genome size was determined by comparison to a fragment size ladder to determine the size of each phage genome.

### 2.5. Lysogeny Detection

Host bacteria were streaked onto a PYE plate from the center of the lysis produced by spotting the phage lysate on the host bacteria. After two days at 30 °C, the colonies from the streak plate were transferred to a velveteen cloth stretched on a circular support and subsequently transferred onto a PYE plate containing 3.5 mL of SSM and 100 μL of either *Caulobacter vibrioides* CB15 or CB13. Lysogenic colonies were identified by the presence of a circle of lysis around the transferred colony. This experiment was independently repeated three times to confirm the consistency and reproducibility of the findings.

### 2.6. Episomal Bacteriophage Genome Detection

Agarose plugs containing bacterial and bacteriophage DNA were prepared following the protocol described by Dingwall et al. [36]. Pulsed-field gel electrophoresis (PFGE) was performed as described above. To detect episomal phage genomes, the region of the bacterial lane corresponding to the expected phage genome size was excised from the gel. The excised gel fragment was placed into a tube containing fresh TE buffer (10 mM Tris, pH 8.0, 1 mM EDTA) and incubated at 4 °C for two weeks to allow DNA diffusion into the buffer. A sample of the buffer was then analyzed by PCR using CbK DNA polymerase primers to confirm the presence of phage genomes within the bacterial cells. This experiment was independently repeated three times to confirm the consistency and reproducibility of the findings.

### 2.7. PCR Analysis of Additional Phage Genomes

Agarose plugs containing phage DNA were prepared using the protocol described by Dingwall et al. [36]. After allowing time for some of the DNA to diffuse out of the plugs, a 1 µL sample of the plug buffer was analyzed by PCR using CBK gp233 and Rogue gp076 and gp077 primers. Ten µL of the amplified DNA from each phage was visualized using conventional gel electrophoresis to confirm successful amplification. The nucleotide sequences of the amplified samples were determined by Eurofins Genomics (Louisville, KY, USA).

### 2.8. Phage Genome Sequencing and Annotation

Phage genomic DNA was prepared from 3–5 mL of a concentrated, high-titer (>10^10^ PFU mL^−1^) phage lysate using a Qiagen DNA isolation kit (Germantown, MD, USA) according to the manufacturer’s instructions. The resulting phage DNA was combined with DNA from other bacteria and phages, and the nucleotide sequences of the combined DNAs were determined using PacBio sequencing technology (Menlo Park, CA, USA) and HGAP4 assembly at the Delaware Bioinformatics Institute or the University of Maryland Institute for Genome Sciences. The nucleotide sequences of the phage genomes were annotated using the Rapid Annotation using Subsystem Technology (RAST) [37], and the annotated sequences were trimmed to remove duplicate sequences at the ends of each phage genome contig using Artemis [38]. The resulting annotated genomes were then compared using Artemis [38] and Mauve [39].

NCBI accession numbers for the genome nucleotide sequences described in this paper include the following: RBC63, PV231352; RBC57, PV231353; RBC8, PV231354; RBC11, PV231355.

### 2.9. Transmission Electron Microscopy

Purified phage isolates were visualized using a JEOL 200CX (JEOL USA, Peabody, MA, USA) transmission electron microscope (TEM) to analyze their morphology. For sample preparation, a formvar/carbon-coated 300-mesh copper grid was carefully floated on a 10 μL drop of a 1:1 mixture of 2% uranyl acetate and phage lysate for 1–2 min to facilitate phage adsorption onto the grid. Excess liquid was gently removed by blotting the edge of the grid with filter paper, and the grid was allowed to air-dry completely at room temperature. TEM imaging was then performed to examine the structural features and morphology of the phage particles.

## 3. Results

### 3.1. Isolation and Characterization of Bacteriophages

**Figure 1:**
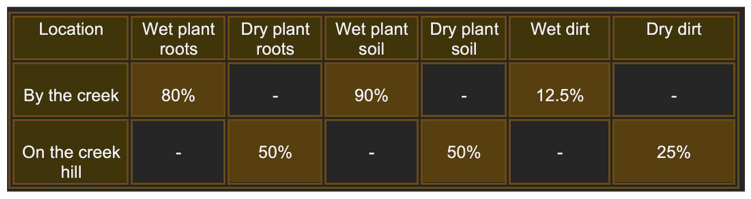
Frequency of isolation of phages from samples taken at the intersection of Rocky Branch Creek and Sumter St. on the University of South Carolina Columbia campus. Each area was sampled 10 times during the course of the year 2022. Percentages represent the successful attempts to isolate bacteriophages.

To gain a deeper understanding of the diversity and biology of bacteriophages and their bacterial hosts in natural environments, we conducted extensive sampling of plant roots and soil at various sites along Rocky Branch Creek on the University of South Carolina campus. The results indicate that plant roots and the accompanying dirt were the most reliable sources of phages, with wet samples better than dry samples (Figure 1). In this study, we characterized 20 new bacteriophages, including 14 additional bacteriophages isolated directly from the environmental samples and independent of their host bacteria.

To identify additional bacteriophages associated with the 21 bacterial strains isolated from the samples described above, we used *Sphingomonas* RBW1, *Caulobacter* strains CBR1 and RBW21 as potential hosts, and identified four new bacteriophages, designated RBC50, RBC67, RBC71 and RBC73, that could infect these hosts (Table 1). We were unable to isolate bacteriophages from the RBW23 bacterial strain even though the presence of a bacteriophage associated with *Caulobacter rhizosphaera* RBW23 was suggested by its ability to lyse a potential phage host strain [33]. Also, *Sphingomonas* strain RBW11 failed to lyse any of our potential host strains even after growth in the presence of Mitomycin C, suggesting that its genome does not contain any intact prophage genomes.

Interestingly, the RBC50 phage was isolated from its host, *Sphingomonas* strain RBW1, and could infect its RBW1 host, indicating that the phage did not provide the typical protective benefits seen in lysogeny. We also characterized two additional bacteriophages, RBC68 and RBC69, that had not been described previously (Table 1).

In addition to isolating bacteriophages from lysogenic bacteria, we isolated 14 new bacteriophages from soil samples using *Caulobacter* SC1004 as a phage-accepting bacterial host strain [40]. These phages were identified based on their ability to form clear plaques of varying sizes, ranging from 0.1 to 1 mm, on SC1004 lawns, indicating a productive lytic infection (Table 2).

### 3.2. Bacteriophage Genome Sizes

**Figure 2:**
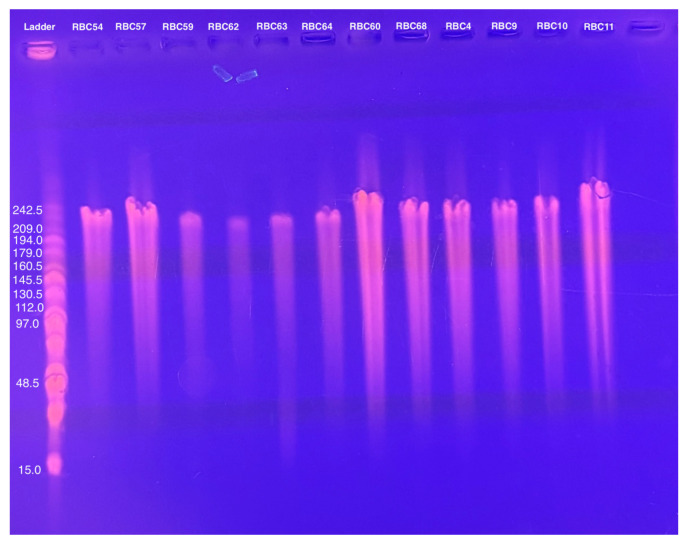
Genome size determination of representative phages using PFGE of purified phage DNA embedded in agarose as described in the Methods.

**Figure 3:**
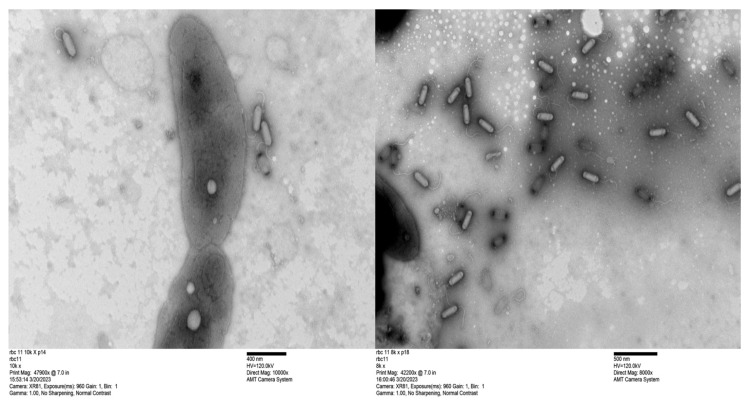
Transmission electron micrographs of bacteriophage RBC11. The scale bar represents 400 nm for the RBC11 in the left image and 500 nm for the RBC11 in the right image with bacterial cells included for a size comparison. Phage capsids with black ends contain no DNA.

The genome sizes of 19 of the 20 new phages were similar to those of the CbK and Rogue phages, ranging from approximately 190 to 240 kbps (Figure 2). This similarity in genome size suggests that these newly isolated phages share structural and functional characteristics with the CbK and Rogue phages since no other known *Caulobacter* phages have genomes in this size range. In contrast, the 300 kb genome size of RBC50 suggests it belongs to one of the other *Dolichocephalovirinae* species that have genomes in the size range. In addition, the morphology of representative phages was determined by electron microscopy and shown to be typical of the CbK and Rogue phages with an elongated head and a flexible tail (Figure 3).

### 3.3. Phage Genomes Are Present in the Cytoplasm of the Host Strains

**Figure 4:**
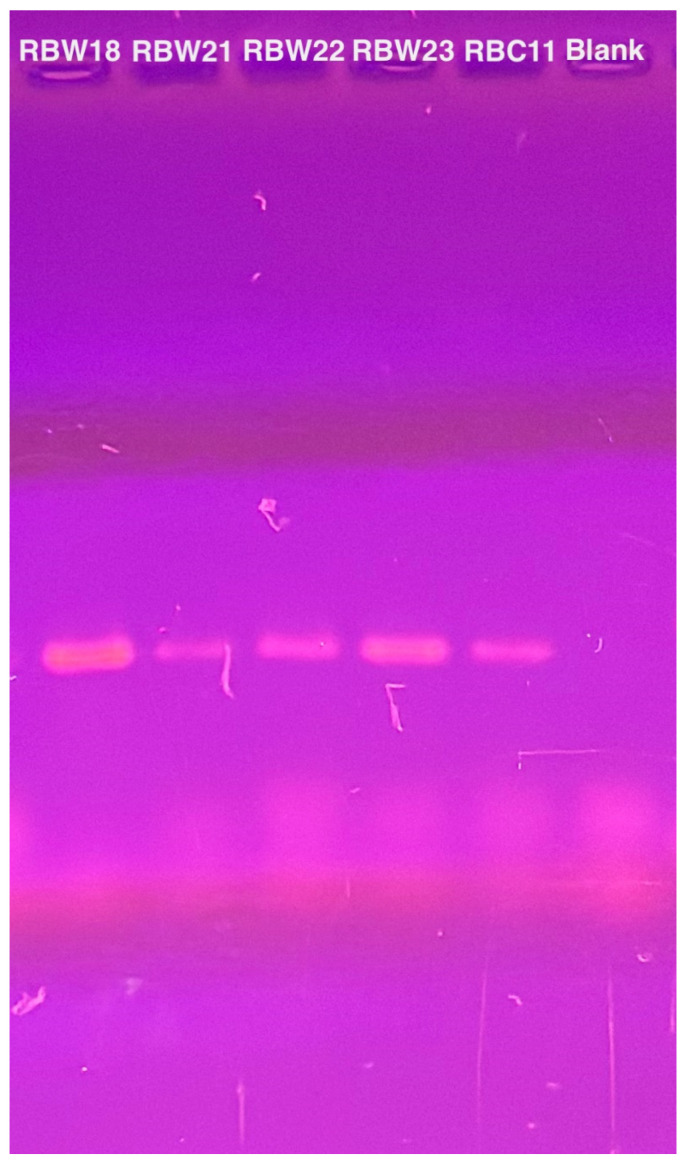
PCR amplification of intact DNA from four bacteria using CbK DNA polymerase primers to detect the presence of phage genomes. DNA from phage RBC11 was amplified as a positive control and the lane labeled blank is the no DNA negative control.

To verify the presence of the phage genomes within the host cells, we performed PCR with CBK DNA polymerase primers to analyze phage DNA from bacterial strains RBW18, RBW21, RBW22, and RBW23, and PCR products were obtained from all four lysogenic strains (Figure 4). Interestingly, although we were unable to isolate a phage from the RBW23 *Caulobacter rhizosphaerae* strain, our results revealed the presence of giant CBK-like phage DNA within the RBW23 strain. Thus, this PCR result confirms that at least a partial phage genome is present in RBW23.

**Figure 5:**
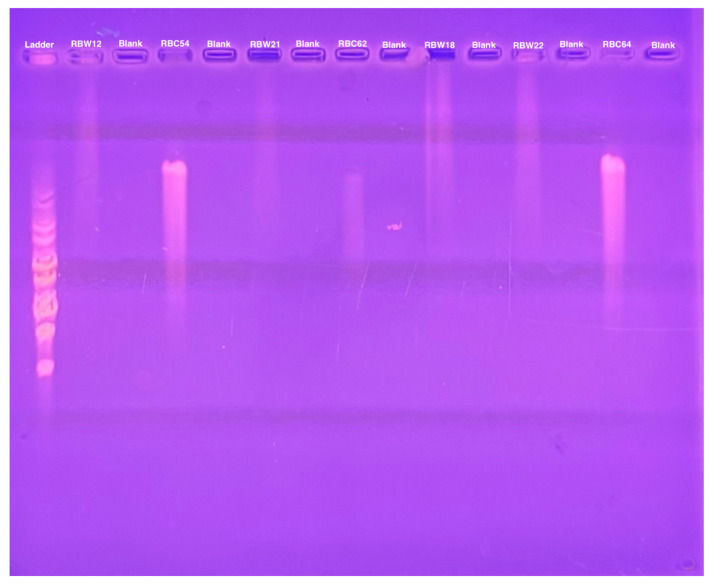
Pulsed field gel electrophoresis of intact bacterial and phage DNA embedded in agarose.

**Figure 6:**
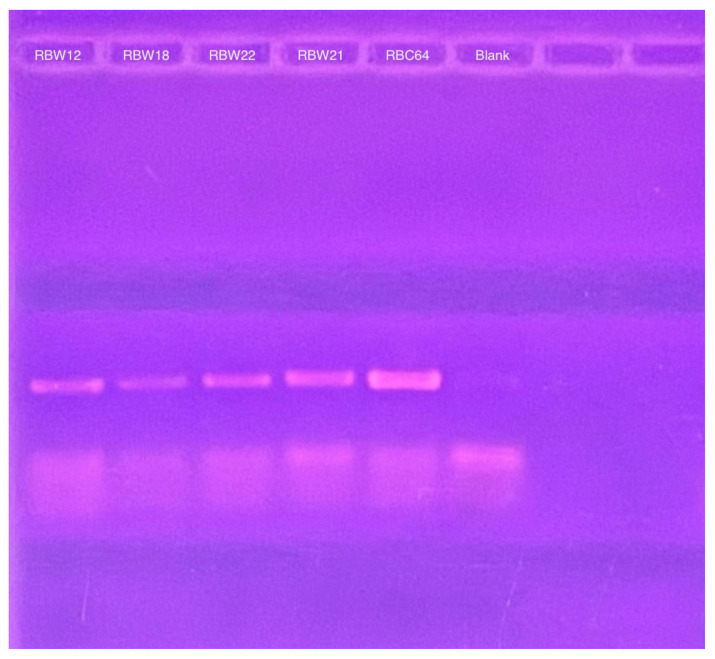
PCR products derived from CbK DNA polymerase primers to detect the presence of cytoplasmic phage DNA in the RBW12, RBW18, RBW22, and RBW21 strains. RBC64 phage DNA was used as a positive control and the lane labeled blank was from a PCR reaction that contained no added DNA as the negative control.

To determine whether these CbK-like phage genomes reside in the cytoplasm of bacterial cells or have been integrated into the bacterial DNA, genomic DNA plugs from four lysogenic bacteria (RBW12, RBW18, RBW21, and RBW22) and three CbK-like phages (RBC54, RBC62, and RBC64) were inserted into the wells of a PFGE gel. Since the bacterial DNA in the plugs consists primarily of intact chromosomes, most of the DNA stays in the wells under these PFGE conditions (Figure 5). In contrast, the much smaller intact phage genomes move well into the gel. Therefore, if the phage genomes in the lysogenic strains had integrated into the host chromosomes, they would remain with the chromosome in the wells of the gel. However, if the phage chromosomes were present as an episome in the bacterial cytoplasm, the episomes would migrate to the position in the gel where the intact phage genomes were located since they would be the same size. Therefore, we excised the region of the bacterial lanes that corresponded to the size of the phage DNA and placed the excised gel fragment into a tube containing fresh plug buffer. After waiting for DNA from the excised piece to diffuse into the buffer, we performed PCR to determine if phage DNA was present. Our PCR analysis revealed that all four lysogenic bacterial strains RBW12, RBW18, RBW21, and RBW22 contained phage DNA at the location of the gel where phage genomes would migrate (Figure 6). Episomal phage DNA was also shown to be present in strains (RBW13 and RBW23) in a similar experiment. Taken together, these results confirm the presence of free phage DNA within the bacterial cytoplasm of the tested strains, confirming that the phage genomes were present as episomes in the host cells. Although the *Dolichocephalovirinae* phage particles contain linear genomes, the genomes contain terminal repeats that would recombine to generate a circular phage genome within the host cytoplasm. Since these phage genomes are stably inherited by both host bacterial daughter cells during each cell division, they must have the ability to replicate as episomes prior to each cell division. The simplest hypothesis would be that the phage episomes contain a sequence that is recognized by the host cell as an origin of replication. Thus, like conventional plasmids, their replication by the host DNA replication machinery would not require any expression of the phage genome.

### 3.4. Genome Nucleotide Sequences

To determine how these new bacteriophages are related to the previously characterized CbK and Rogue phages, we amplified the phage DNA with CbK gp233 primers and also with primers that span the Rogue gp076 and gp077 genes. The DNA from all 14 bacteriophages from soil (Table 1), as well as RBC54, RBC58, RBC62, RBC63, RBC64, and RBC65 [33] and RBC68 (Table 2), was successfully amplified with the Rogue primers, but not with the CbK gp233 primers. Thus, these phages belong to the *Poindextervirus rogue* species. In contrast, DNA from phages RBC57, RBC60, and RBC61 were amplified with the CbK gp233 primers, but not with the Rogue primers, so they are members of the *Shapirovirus CbK* species.

**Figure 7:**
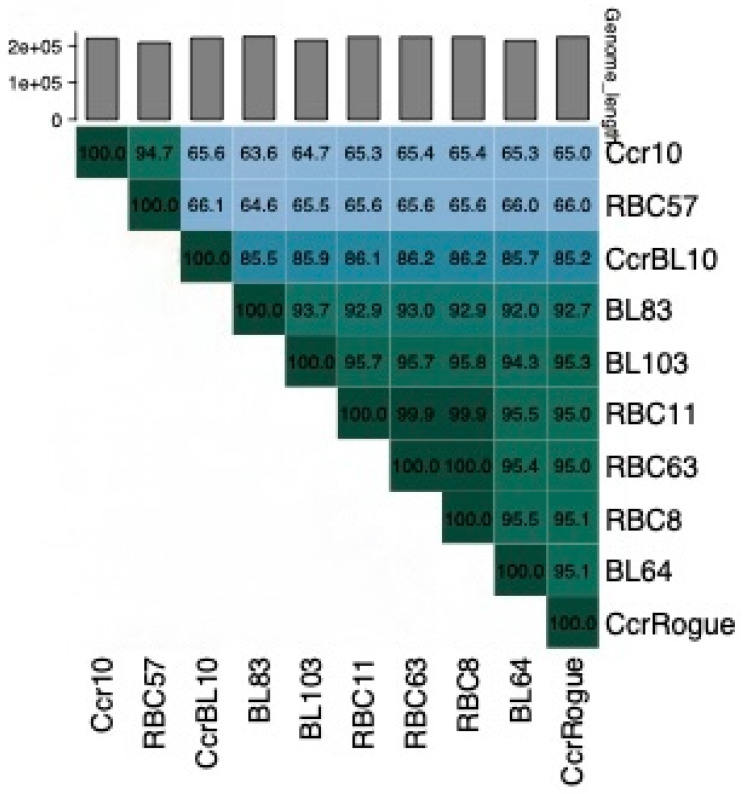
A Viridic analysis [41] of 11 giant phage DNAs. Ccr10 is a representative of the *Shapirovirus CbK* species. CcrBL10 is a representative of the *Poindextervirus BL10* species and CcrRogue, BL64, BL83, and BL103 are representatives of the *Poindextervirus rogue* species [42].

The genome nucleotide sequences of four of these phages, RBC8, RBC11, RBC57, and RBC63, were determined and then compared using the Viridic program [41]. This comparison revealed that the RBC57 genome is closely related to that of phage Ccr10 (Figure 7), indicating that it belongs to the *Shapirovirus* genus. In contrast, the RBC11, RBC8, and RBC63 genomes are closely related to that of the Rogue phage, verifying that they are members of the *Poindextervirus rogue* species.

## 4. Discussion

Bacteriophage diversity plays a crucial role in shaping bacterial communities and influencing microbial interactions in various ecosystems. In this study, we expanded our understanding of bacteriophages isolated from soil and plant roots along Rocky Branch Creek at the University of South Carolina. Previously, 21 bacterial strains were isolated from this environment, with 20 out of 21 identified as lysogenic. Further investigation led to the isolation of 18 bacteriophages from these lysogenic bacteria using *Caulobacter* strains SC1004 and CB13 [33]. In addition, in this study, 14 more bacteriophages were isolated from soil samples from the same location along with four additional phages from lysogenic bacteria associated with different bacterial strains. Notably, all these phages are giant phages with genome sizes ranging from 190 to 240 kb and belong to the CBK-like *Dolichocephalovirinae* subfamily [42]. The presence of *Dolichocephalovirinae* phages in this ecosystem suggests their essential role in microbial community interactions. Their association with a variety of bacterial strains highlights their potential ecological significance. While previous studies of agricultural soils have identified diverse bacteriophages that are not closely related and typically exhibit narrow host ranges [43,44,45,46,47,48], our study isolated closely related phages from both diverse bacterial hosts and directly from soil and plant roots. Given their abundance and association with a wide variety of soil bacteria, these phages may contribute to horizontal gene transfer, bacterial population dynamics, and microbial adaptation in plant-associated environments.

The isolation of this collection of closely related bacteriophages from a broad range of bacterial hosts suggests that these phages are shared among many kinds of bacteria within the soil community. Understanding the dynamics of this microbial community is a key area of interest, and our lab plans to investigate these interactions further in future studies. These findings highlight the complexity of phage–bacteria interactions and suggest that phage-mediated bacterial dynamics are more intricate than previously thought. Future studies will focus on characterizing the broader host range of CBK-like phages, examining the molecular basis of their interactions with both Gram-positive and Gram-negative bacteria, and exploring how these phages influence bacterial survival and adaptation in natural environments.

Another key discovery of this study is the unique lifecycle of these giant phages. While classical lysogeny involves phage genome integration into the bacterial chromosome [49], our experiments revealed that these phages remain free in the bacterial cytoplasm yet retain full infectivity, actively replicating their genomes without requiring integration into the host genome. Unlike some pseudolysogenic phages, which remain dormant under nutrient deprivation and reactivate under favorable conditions, we demonstrated that these *Dolichocephalovirinae* phages persist in the cytoplasm as episomal pseudolysogens. Further research is required to elucidate the molecular mechanisms governing episomal lysogeny, its impact on host physiology, and its potential applications in biotechnology and phage therapy. Understanding how these phages influence microbial communities and plant-associated bacteria may provide valuable insights into their role in shaping ecosystem dynamics. Unraveling these interactions could provide valuable insights into their ecological roles and potential applications in agriculture and biotechnology.

In addition, the discovery of RBC50 was particularly unusual, not only due to its isolation from its *Sphingomonas* host (RBW1) but also because of its unexpected behavior within the host. In classical lysogeny, prophages integrate into the bacterial genome and provide immunity against superinfection by the same or closely related phages [23,24,26]. However, RBC50 defies this paradigm by remaining infectious to its host, suggesting a more complex and unconventional interaction between the phage and bacterial cell.

One likely explanation is that RBC50 does not establish true lysogeny but instead exists as a pseudolysogen. In this state, the phage genome persists episomally within the host without integrating into the bacterial chromosome, similar to other giant phages discussed in this study. Unlike classical lysogens, these episomal pseudolysogenic phages may not produce the repressor proteins required for superinfection immunity. Thus, RBC50, along with the five other phages identified in our previous study [33], can form plaques on a host strain that already contains an episomal copy of the infecting phage genome.

These findings challenge conventional models of lysogeny by demonstrating that not all prophages provide superinfection immunity. Instead, they highlight the diverse strategies phages employ to coexist with their hosts, shedding light on the intricate balance between lysogeny, pseudolysogeny, and lysis. These insights have broader implications for microbial ecology, particularly in understanding the regulation of bacterial populations in natural environments.

## 5. Conclusions

This study emphasizes the importance of targeted sampling in natural settings for uncovering phage–host interactions that may be unique to specific ecological niches. Our key findings highlight that 14 phages isolated independently from the soil and 22 phages isolated from diverse lysogenic bacteria from different sites of Rocky Branch Creek are closely related. These bacteriophages belong to the *Dolichocephalovirinae* subfamily and are capable of persisting as episomal pseudolysogens within various soil bacteria, revealing a complex and dynamic interaction between phages and their hosts. Furthermore, we demonstrated that these phages could infect their pseudolysogenic hosts, which remain susceptible to them, thus challenging conventional models of lysogeny and superinfection immunity.

This study also suggests a critical ecological role of these shared bacteriophages in shaping bacterial communities, influencing plant–microbe interactions, and regulating microbial processes essential for soil health and plant growth. Our findings open new avenues for understanding the intricate relationships within the soil microbiome, and they highlight the potential applications of bacteriophages in sustainable agriculture and microbial ecology. Ultimately, this research enhances our understanding of the diverse strategies phages employ in the environment and their potential to support soil and plant health.

The future directions of this study will involve testing the host range of these phages on a broader spectrum of bacterial strains to better understand their potential applications in microbial ecology and phage therapy. Our laboratory will also investigate the potential of these phages in agricultural settings, including their role in controlling plant pathogens and promoting plant growth. By expanding our understanding of these phages, we hope to develop more targeted and sustainable strategies for microbial management in soil environments.

## Figures and Tables

**Table 1:** Characteristics of six novel giant bacteriophages associated with soil bacterial strains.

Bacteriophage Name	RBC50	RBC67	RBC68	RBC69	RBC71	RBC73
Original Source	RBW1 Sphingomonas frigidaeris	RBW6 pseudomonas	RBW26 Caulobacter sp.	RBW29 Caulobacter vibrioides	RBW18 Brevundimonas	RBW25 Caulobacter rhizosphaerae
New host strain	RBW1	RBW21	SC1004	CB13	CBR1	CBR1
Plaque Morphology	Clear	Clear	Clear	Clear	Clear	Clear

**Table 2:** Bacteriophages isolated from Rocky Branch Creek soil.

Bacteriophage Name	RBC4	RBC7	RBC8	RBC9	RBC10	RBC11	RBC12	RBC15	RBC17	RBC18	RBC22	RBC29	RBC31	RBC32
Date collected	10/21	12/21	12/21	1/22	1/22	1/22	1/22	1/22	3/22	3/22	3/22	3/22	3/22	4/22

## Data Availability

The original contributions presented in this study are included in the article material. Further inquiries can be directed to the corresponding author.
